# The Effects of* Agave fourcroydes* Powder as a Dietary Supplement on Growth Performance, Gut Morphology, Concentration of IgG, and Hematology Parameters in Broiler Rabbits

**DOI:** 10.1155/2016/3414319

**Published:** 2016-09-29

**Authors:** Maidelys Iser, Yordan Martínez, Hengjia Ni, Hongmei Jiang, Manuel Valdivié Navarro, Xiaosong Wu, Naif Abdullah Al-Dhabi, Manuel Rosales, Veeramuthu Duraipandiyan, Jun Fang

**Affiliations:** ^1^Hunan Province University Key Laboratory for Agricultural Biochemistry and Biotransformation, Hunan Agricultural University, Changsha, Hunan 410128, China; ^2^Hunan Co-Innovation Center for Utilization of Botanical Functional Ingredients, Changsha, Hunan 410128, China; ^3^Institute of Subtropical Agriculture, Chinese Academy of Sciences, Changsha, Hunan 410125, China; ^4^Centro de Estudios de Producción Animal, Universidad de Granma, Apartado Postal 21, Bayamo, 85100 Granma, Cuba; ^5^Instituto de Ciencia Animal, Apartado Postal 24, San José de Las Lajas, Mayabeque, Cuba; ^6^Department of Botany and Microbiology, Addiriyah Chair for Environmental Studies, College of Science, King Saud University, P.O. Box 2455, Riyadh 11451, Saudi Arabia; ^7^Centro de Investigación de Patología Animal, Departamento de Medicina Veterinaria, División de Ciencias Veterinarias, Centro Universitario de Ciencias Biológicas y Agropecuarias, Universidad de Guadalajara, Guadalajara, JAL, Mexico

## Abstract

This study was conducted to determine the effects of* Agave fourcroydes* powder as a dietary supplement on the growth performance, gut morphology, serum concentration of IgG, and the hematology parameters of broiler rabbits. A total of 32 rabbits [New Zealand × Californian] were weaned at 35 days. They were randomly selected for two dietary treatments (eight repetitions per treatment), which consisted of a basal diet and a basal diet supplemented with 1.5% dried-stem powder of* A. fourcroydes*. On day 60 from the initiation of treatment, gut histomorphology (duodenum and cecum), serum concentration of IgG, and hematology parameters were all measured. The results showed that* A. fourcroydes* powder supplementation improved (*P* < 0.05) the ADFI, ADG, and final BW. Correspondingly, this treatment increased (*P* < 0.05) the muscle and mucosa thickness and height and width of villi. However, duodenum crypts depth was lower (*P* < 0.05) when rabbits were fed with this natural product, compared with the basal diet treatment. Results also indicated that the* A. fourcroydes* powder increased (*P* < 0.05) the serum concentration of IgG but did not change the hematology parameters. This data indicates that* A. fourcroydes* powder, as a supplement, had beneficial effects on increasing the growth performance and serum concentration of IgG, as well as improving the gut morphology without affecting the hematology parameters in broiler rabbits.

## 1. Introduction

The constant antibiotic applications, as additives for animal feed, could provoke an increase of the number of resistant strains, as well as an immense risk of crossed resistance spreading to other microorganisms [1]. Although antibiotics have been used to a lesser extent in rabbits, compared with poultry and pigs, the use of antibiotics as zinc-bacitracin has been applied to reduce the proliferation of* Clostridium perfringens* and other pathogens, mainly after weaning [2].

In the interests of public health, especially the prevention of the indiscriminate use of dietetic antibiotics, which have been the subject of worldwide concerns, some antibiotic-alternatives that have had positive effects on growth performance and product quality should be used in the diets of nonruminant animals [3]. Natural products are considered better alternatives to Antibiotic Growth Promoters (AGP), from the point of view of biosafety and low residue [4].

The* Agave* genus, a part of the Agavaceae family, is native to Mexico. They have been cultivated since the pre-Columbian era for the production of textile fibers, alcohol, molasses, pulp, and fodder, as well as for erosion control and soil conservation [5]. Specifically, the stem of the* Agave fourcroydes* has a high oligosaccharide content, which forms a polydisperse mixture [6]. Also, our previous studies have demonstrated the presence of flavonoids, anthocyanins, saponins, coumarins, reducing sugars, and tannins in this plant material [7].

On the other hand, the dried-stem powder of* Agave tequilana*, which are rich in fructans type inulin, used as a supplement in the diets of swine, enhances immunity, microbiology, and intestinal morphology, as well as promoting the growth performance and meat quality [8]. Other studies have also shown that using extracts of* A. tequilana* and* A. fourcroydes* with the diets of mice decreased the cholesterol and glucose serum levels, as well as increasing the production of volatile fatty acids and the growth of lactic acid bacteria [6, 9].

There are many chemical benefits of* Agave *spp. for animal feed. However, there have been few studies done on the dietary use of dried-stem powder of* A. fourcroydes* in animal nutrition, especially on the diet of rabbits. It can be hypothesised from previous studies that dietary supplementation with this natural product may have the potential capability of stimulating the immune system and modulating the intestinal integrity, thereby improving the growth performance in rabbits. Thus, the objective of the current study was to evaluate the effects of dried-stem powder of* Agave fourcroydes *on growth performance, gut morphology, serum concentration of IgG, and hematology parameters of broiler rabbits.

## 2. Materials and Methods

### 2.1. Animal, Housing, and Treatment

This study was carried out in accordance with the Mexican guidelines for animal welfare and experimental protocol, which is approved by the Animal Care Committee, University of Guadalajara, Mexico. A total of 32 rabbits [New Zealand × Californian] that were weaned at 35 days with an initial BW of 768 to 769 g were randomly selected to two dietary treatments.

The dietary treatments consisted of a basal diet (BD) and a basal diet supplemented with 1.5% dried-stem powder of* A. fourcroydes*. There were 16 rabbits in each treatment, with two rabbits per pen. The pen is 76-cm long, 76-cm wide, and 45-cm high.

Feed and water were freely available during the entire experimental period, which lasted for 60 days. The temperature was kept at 22 (±2)°C, and relative humidity was maintained between 60 and 65%. BD was prepared according to the nutritional requirements of broiler rabbits [11]. Ingredients of the diets are summarised in Table 1. The dried-stem powder of* Agave fourcroydes* was kindly provided for the study by the Study Center of Animal Production, Faculty of Veterinary Medicine, University of Granma, Cuba.

### 2.2. Growth Performance

Rabbits were weighed (BW) on days 35 and 95. Feed intake (g/rabbit/day) was measured daily. Average daily gain (ADG), average daily feed intake (ADFI), feed/gain ratio (F/G), and viability were calculated for the period of 1 day to 60 days during the trial.

### 2.3. The Analysis of Gut Morphology

At the end of the experiment, rabbits (one rabbit/pen) were killed to sample gut tissues. The gastrointestinal tract (GIT) was divided into two segments: duodenum and cecum. Approximately 5 cm of intestinal tissue was cleaved, removed, and fixed at 10% formalin in PBS, at 4°C for histomorphological analysis.

The formalin-fixed samples, duodenum and cecum tissues were initially dehydrated in a graded series of ethanol. These light microscopic observations were followed by the samples being embedded in paraffin wax. Then, the tissues were sectioned at 5 *μ*m thickness and mounted in slides. After dewaxing, hydrating, and staining the tissues with Hematoxylin-Eosin, the thickness of muscle and mucous membrane, the width and depth of the crypts, and the height and width of villi of the duodenum and cecum were determined by using an Axiostar microscope (Carl Zeiss, Oberkochen, Germany) connected to a computer with Analysis-Opti Basic and soft imaging system software. Images to 500x and 100x were obtained [12]. Then, the villus height/crypt depth ratio was calculated.

### 2.4. The Determination of Hematology Parameters and Serum Concentration of IgG

Blood samples were collected from the jugular vein of eight rabbits, one rabbit per pen per treatment, on the day of euthanasia at 95 days old; one part of the samples was collected in 20 mL tubes and stood for one hour. Then, the serum was separated by centrifugation at 10,000 rpm for 25 minutes, at 20°C, using Eppendorf centrifuge 5804, USA. The other blood sample (whole blood) was placed in 2 mL tubes with sodium heparin, which was added at a ratio of 2 : 1. Both samples were stored at −20°C until analyses in the laboratory.

Leukocytes were analysed by blood smear and Giemsa dye. The hemoglobin was analysed by the HemoTest. Hematocrit was determined according to Wintrobe. Total proteins were measured by Biuret using spectrophotometer Shimadzu UV-Visible 160 A (Japan). Erythrocytes and platelets were analysed by cell counting using a Neubauer chamber and the automatic platelets counter, respectively.

The Concentration of the Mean Corpuscular Hemoglobin (CMCH), the Mean Corpuscular Hemoglobin (MCH), and the Mean Corpuscular Volume (MCV) were determined by the following formulas:

(i) CMCH = Hb (g/100 mL) × 100/Ht (%).
(ii) MCH: (Hgb *∗* 10)/leukocytes.
(iii) MCV: Ht (%) *∗* 10/RBCs (millions/mm^3^).

The serum concentration of IgG was determined using a commercially available 125I Radio Immunoassay analyser kit with *γ*-calculating instrument GC-300 (Beijing, China), according to the manufacturer's instructions. The kits are commercially available from Kemeidongya Biotechnology Company (Beijing, China).

### 2.5. Statistical Analysis

Data was subjected to analysis of variance (ANOVA) for simple classification of completely randomised design. Prior to the analysis of variance, the normality of the data and the uniformity of variance were verified by Kolmogorov-Smirnov and the Bartlett test, respectively, using the statistical software SPSS version 17.1.

## 3. Results 

### 3.1. Growth Performance

All rabbits were healthy and grew well throughout the entire experimental period of 60 days.* A. fourcroydes *powder supplementation improved (*P* < 0.05) the final BW, ADG, and ADFI compared with BD (Table 2). However, F/G did not show significant differences (*P* > 0.05) among treatments.

### 3.2. Gut Morphology

Table 3 illustrates the data from the analysis of the gut morphology of broiler rabbits at 95 days old. In the duodenum and cecum, the* Agave fourcroydes* powder increased (*P* < 0.05) the muscle and mucosa thickness compared with BD, as well as improving the (*P* < 0.05) height and width of villi. However, the duodenum crypts depth of* A. fourcroydes*-treated group was lower (*P* < 0.05) than that of BD group (Table 3). Meanwhile, the width and depth of cecum did not show significant differences (*P* > 0.05) amongst the treatments.

### 3.3. Hematology Parameters and Serum Concentration of IgG

**Figure 1:**
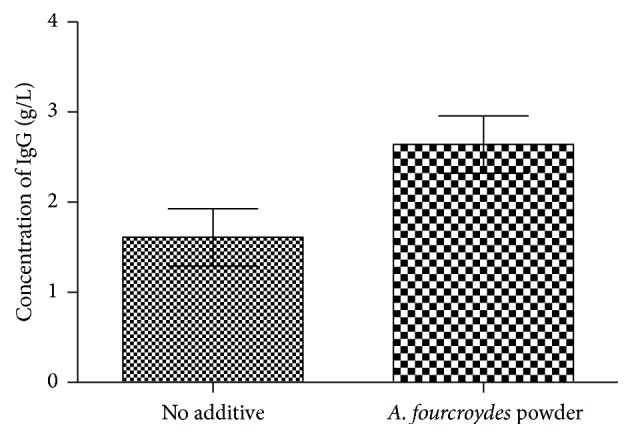
Effect of dietary supplementation of 1.5%* Agave fourcroydes* powder on concentration of IgG of broiler rabbits (95 days old) (SEM ± 0.319; *P* value = 0.049). The experiment lasted for 60 days; *n* = 8.

Dietary supplementation with* A. fourcroydes* powder did not influence (*P* > 0.05) the hematology parameters of broiler rabbits according to Table 4. It can also be seen that these parameters of* A. fourcroydes*-treated rabbits were in the normal range according to previous reports [10]. In addition, the serum concentration of IgG was higher (*P* < 0.05) when rabbits were fed with the* A. fourcroydes* powder as feed additives (Figure 1) compared with the BD group.

## 4. Discussion 

The viability (100%; data not shown) was excellent for both treatments, which indicated the innocuousness of the product for a 60-day course. The present results showed that* A. fourcroydes* powder supplementation at 1.5% led to a higher BW compared with the BD group, suggesting that this material may contain some compounds that could enhance the growth performance of the rabbits.

Fructans, which are recognized as one kind of oligosaccharides presented in the* A. fourcroydes, *are not digestible by the digestive enzymes of the host but can be metabolised by microorganisms in the large intestine, which is beneficial for the synthesis of short-chain fatty acids. Thus,* A. fourcroydes *is capable of positively affecting the growth performance by increasing the production of short-chain fatty acids [13, 14].

In rabbits, fructans were reported to increase the population of lactic acid bacteria, particularly* Lactobacillus* spp. and* Bifidobacterium* spp., which provokes a competitive exclusion against pathogenic bacteria in the GIT, as well as a beneficial influence on body weight [15]. This study coincides with the results of Abdel-Aziz et al. [16], which reaffirms the importance of* Lactobacillus* spp. in the efficiency of the digestive process.

Also, this natural product has a high presence of flavonoids, a polyphenolic compound with the ability to inhibit the production of nitric oxide (NO), interleukin-6 (IL-6), and prostaglandin E2 (PGE2) in LPS-induced macrophage cells [17], which ensures improvement in the absorption of nutrients, subsequently increasing the body weight [18, 19].

In addition, other secondary metabolites detected in* A. fourcroydes* powder, such as tannins, reducing sugars, anthocyanins, and saponins [7], could have beneficial effects on growth performance, especially for the control of pathogenic microflora and the modulation of innate and humoral immune response [3, 20]. Some studies have found a positive relationship between the incorporation of small concentrations of beneficial secondary metabolites in diets and productive behavior [21, 22].

However, other studies have found that the extracts of* Agaves* (*tequilana* and* fourcroydes*) were unable to increase the growth performance of laboratory mice [6, 9]. It is possible that the effects of products rich in fructans on body weight depend on the concentration of fructans in the diet, as well as the animal species under study.

An important discovery in this study is that the supplementation of* A. fourcroydes* increases the thickness of muscle and mucosa in broiler rabbits and subsequently improves intestinal health [12]. The competitive exclusion in the GIT reduces the adhesion of pathogenic bacteria, which consequently results in a decrease in intestinal lesions and has a positive effect on the thickness of these layers [23]. In our previous studies, using the* A. fourcroydes* powder as a supplement increased the proliferation of lactic acid bacteria, as well as decreasing cecal pH [7].

de Blas et al. [23], studying the relationship between microorganisms and intestinal health, found a marked reduction of mucosa thickness induced by the proliferation of* C. perfringens*,* Campylobacter* spp., and* Helicobacter* spp., which in turn has a negative impact on the viability and biological responses of rabbits. It is well known that the intestinal layer plays an important role in the defense against infectious diseases of the host. The improvement of an intestinal barrier function, though modulating the intestinal microbial community diversity, is mostly beneficial for the animal's health [24]. Furthermore, Raj et al. [25] found that intestinal permeability is directly related to the thickness of the intestinal mucosa. They found that a reduction of the intestinal mucosa would lead to the uncontrolled access of toxins, chemicals, microorganisms, and macromolecules, which could cause enteric problems and reduction of growth performance [23].

In addition, the mucosal thickness of duodenum is greater than that of cecum, due to its great absorptive capacity. However, the muscle thickness of cecum is more beneficial in transporting the large and heavy feces in rabbits due to caecotrophy [26].

Similarly, Jiang et al. [12] reported that the morphology of the villi and crypts has been associated with bowel function and growth performance of animals. Our results are consistent with these findings, as it was observed that* A. fourcroydes* powder supplementation (1.5%) increased (*P* < 0.05) the height and width of the villi, due to suitable intestinal conditions. These conditions include higher proliferation of lactic acid bacteria, decrease of cecum pH [7], and thickening of the intestinal mucosa, which denoted a more mature tissue.

Other studies with diets rich in fructans found similar results to the structure of the villi [27, 28], suggesting that there may indeed be an association between the intestinal health status and the absorptive capacity. However, Mourão et al. [29] found no significant effects of fructooligosaccharide on the intestinal structure in rabbits (*P* > 0.05).

Understanding the relationship between villi and crypt is helpful, in order to estimate the nutrient digestion and absorption capacity of the small intestine [30]. The higher the ratio of villi/crypt, the better efficiency there is in the digestive process [31]. In addition, a reduction of villi and crypt results in less absorption cells and more secretory cells [32]. From this point of view, dietary supplementation of* A. fourcroydes* powder can be beneficial in promoting a better productive response (Table 2) through upregulating (*P* < 0.05) the ratio of villi/crypt (5.29) related to the control treatment.

Furthermore, rabbits from the BD group had a higher width and depth of crypts than those from the* A. fourcroydes* group. It is reported that the migration of specialised cells to the villi, especially with the decrease of villus height, would elevate the depth of crypts [33]. However, other studies that use mannan-oligosaccharides as growth promoters did not find any significant changes in the intestinal morphology [26]. However, there is no doubt that the gut can undergo rapid epithelial renewal by shortening the crypt depth [34].

Hematological parameters are currently used as indicators of health in humans and animals. Variations of these indicators may reflect bacterial, viral, parasitic, or fungal infections, as well as intoxication, dehydration, or blood clotting problems [10].

Usually, it is necessary to determine whether the newly introduced feed additive would induce the comprehensive immune response in a body. Some compounds, such as nonenzymatic feed additives, may induce changes in polymorphonuclear leukocytes (neutrophils and eosinophils), mainly by activating the immune system to eliminate the exogenous material and/or the possible toxic and allergenic compounds [35, 36]. Thus, the body's immune response to* A. fourcroydes* powder was studied. After the 60-day treatment, it was found that* A. fourcroydes* powder, with high fructan concentrations and secondary metabolites, did not cause adverse symptoms or diminish the defenses (white blood cells) in rabbits.

The supplementation of low concentrations of* A. fourcroydes* powder did not influence the absorption of iron, as illustrated by the value of hemoglobin in Table 4. This is possibly because this additive did not give rise to any excess of tannins, which could inhibit the absorption of this mineral and induce iron deficiency, causing anemia [36]. On the other hand, the hematocrit value suggests that the animals were under suitable conditions of hydration. Once the hemoconcentration occurred due to water deficit, the hematocrit value would increase [37].

The amount of serum antibody is an indicator of humoral immunity in all mammals [38]. The rabbits can generate abundant antibodies or proteins and promote the proliferation of B lymphocytes to defend against any parasitic or pathogenic infections [39]. Curiously, IgG represents about 80% of the immunoglobulins in serum, which participated in humoral immunity against bacteria and pathogens [20, 40]. Thus,* A. fourcroydes* powder supplementation may improve antitoxic and antibacterial immune responses through the elevation of serum concentration (*P* < 0.05) of IgG in rabbits.

Several studies have shown that certain functional foods can improve the phagocytic activity of the intestinal leukocytes, as well as promoting the proliferation of leukocytes B and secretion of immunoglobulins A and G [41]. Other studies on feed additives in nonruminants have also found similar results when animals were fed with foods rich in secondary metabolites [20, 40, 42].

In summary, feeding with 1.5% of* A. fourcroydes* powder improved the growth performance, as well as the serum concentration of IgG and gut morphology, in broiler rabbits. Supplementation of this product did not affect the hematology parameters, suggesting that it can be used safely as a food additive at a dose of 1.5% for broiler rabbits.

## Figures and Tables

**Table 1:** Compositions and nutrient levels in basal diets (as-fed basis).

Ingredients	Content (%)
Alfalfa hay	12.00
Wheat straw	17.5
Barley grain	19.00
Wheat bran	24.00
Sunflower meal, 30% CP	12.00
Soybean meal, 44% CP	11.0
Soybean oil	2.88
Sodium chloride	0.50
Monocalcium phosphate	0.50
HCL-lysine	0.09
L-Threonine	0.08
DL-Methionine	0.05
Premix^1^	0.50
*Calculated composition*,* % as fed*	
Crude protein	16.70
Digestible energy (MJ/kg)	9.92
Neutral detergent fibre	29.10
Lysine	0.77
Methionine + cystine	0.59
Threonine	0.65
Ashes	5.37

^1^Each kg contains vitamin A 12,000 IU, vitamin D3 2,000 IU, vitamin B2 4160 IU, niacin 16 700 IU, pantothenic acid 8200 IU, vitamin B6 3,420 IU, folic acid 0.980 g, vitamin B12 16 mg, vitamin K 1560 IU, 16 g vitamin E, 8.5 g BHT, 0.750 g cobalt, copper 3.5 g, 9.86 g iron, manganese 6.52 g, 0.870 g sodium, 4.24 g zinc, and selenium 6.67 g.

**Table 2:** Effects of dietary supplementation of *Agave fourcroydes* powder on growth performance of broiler rabbits (95 days old).

Items	Treatments		SEM ±	*P* value
	No additive	1.5% *A. fourcroydes* powder		
Finish BW, g	2398.38	2462.31	12.507	0.001
ADFI, g/d	119.91	123.12	0.625	0.001
ADG, g/d	27.16	28.22	0.285	0.013
F/G	4.42	4.37	0.042	0.461

The experiment lasted for 60 days; *n* = 16.

(i) BW: body weight.

(ii) ADFI: average daily feed intake.

(iii) ADG: average daily gain.

(iv) F/G: feed/gain ratio.

SEM: standard error of the mean.

**Table 3:** Effects of dietary supplementation of *Agave fourcroydes* powder on gut morphology of broiler rabbits (95 days old).

Items, *µ*m	Treatments		SEM ±	*P* value
	No additive	1.5% *A. fourcroydes* powder		
*Duodenum*				
Muscle thickness	117.12	154.80	5.757	<0.001
Mucosal thickness	1070.40	1351.52	32.83	<0.001
Height of villi	892.72	1027.96	26.07	<0.001
Width of villi	108.04	142.48	7.310	<0.001
Crypts depth	98.56	71.68	7.417	<0.001
Width depth	66.92	63.93	4.082	0.424
Villus height/crypt depth	9.06	14.35	1.102	<0.001
*Cecum*				
Muscle thickness	284.00	335.60	30.06	<0.001
Mucosal thickness	426.56	438.20	3.370	<0.001
Crypts depth	243.36	204.20	11.40	<0.001
Width depth	121.48	86.52	6.890	<0.001

The experiment lasted 60 days; *n* = 8. SEM: standard error of the mean.

**Table 4:** Effects of dietary supplementation of *Agave fourcroydes* powder on hematology parameters of broiler rabbits (95 days old).

Items	Treatments		SEM ±	*P* value	Reference values^*∗*^
	No additive	1.5% *A. fourcroydes powder*			
RBCs, millions/mm^3^	6.53	5.92	0.492	0.178	4.5–7.0
Leucocytes, thousands/mm^3^	6.69	6.00	0.476	0.150	6.00–9.30
Hemoglobin, g/dL	13.44	12.70	0.578	0.392	8–15
Hematocrit, %	40.50	37.58	1.814	0.288	30–50
MCH, pg	20.80	19.94	1.321	0.658	19–30
MCV, fL	62.14	58.80	3.613	0.532	40–80
MCHC, g/dL	33.16	33.20	0.076	0.720	32–38
Platelets, thousands/mm^3^	449.21	410.02	15.84	0.118	400–700
Total protein, g/dL	7.28	7.30	0.082	0.867	5.2–7.8

The experiment lasted for 60 days; *n* = 8. ^*∗*^Giusti et al. [10].

(i) MCH: Mean Corpuscular Hemoglobin.

(ii) MCV: Mean Corpuscular Volume.

(iii) MCHC: Mean Corpuscular Hemoglobin Concentration.

SEM: standard error of the mean.
